# Supplementary material for: Genome-Wide Identification and Expression Analysis of ANS Family in Strawberry Fruits at Different Coloring Stages
Source: Int J Mol Sci. 2023 Aug 8;24(16):12554. doi: 10.3390/ijms241612554 (PMC10454780; doi:10.3390/ijms241612554)
Supplement: Supplementary file 1 [file ijms-24-12554-s001.zip › ijms-2439427-supplementary.pdf]

Supplementary Table S1. Primer sequences of *FvANS* gene family used for qRT-PCR.

gene	Forward primer(5′-3′)	Reverse primer (5′-3′)
FvANS1	ACGGGTTTCAGGTATGGAGCAATG	TGTAGGTTGAGCCAGTTGTGAAGC
FvANS4	GGTGTCCAAGAGCCGATAAGGTG	TGTCCTGCAACAGAACAGTCATTCC
FvANS5	GCCTCCTCACACCATCCTCAATC	TCTTCTTCTCTTCCGCTGGCATTG
FvANS9	GAGCATGGCGAGGTCATTGAATTTG	TGAATGTGGCTTGAATCCGAGGATG
FvANS10	AGGAAGGGATCGGCTGGATGTC	CGCACGGTGAATTGTAGTCTTGAAC
FvANS16	GAGAAGGAGGCATTGACGAAGGC	GTGTGGCGTTTGAGTCCGAGAG
FvANS17	ACCTCCTGTCCCTGGAACATTCG	GTCCAAAGGCTCCACTGCTGTATC
FvANS19	GAGTGTTGGACGAGATGCTGGAG	TCAGGCTCAGGACATGGAGGATAG
FvANS26	TCAGAGAGGTATGCCAGGAGTATGC	GCCAACGCCTAGAGCCAAGTG
FvANS31	TTAAGCGGTATCTGGAGGAGGTGAG	ATGGCGGATAGTGATTTGACAGCAG
FvANS32	AGCCTGTCCCTAATGCCTTGGTAG	AAGGATGGATGAAGCTCGCAACTG
FvANS38	GGTGCTTCAGAGCGAGGTCATTG	GCCGAGGTCCGTATGCTTAGTTC
FvANS39	TCACCACCGTCCCTAACACAGTC	TCCACCCACCCTTTCTTCCCTTC
FvANS42	GCTGAGAGGGTAATGGAAGTGATGG	GGGTTGAGGACACGGTGGGTAG
FvANS43	AGGTGGACCGAGGAACTGATGAG	CTGTGTCACTTGGTGCGAGGTAAC
FvANS46	ACTATGGGCTCACTCCTCACACTG	ACCTTCTAGCGGACTTCCAAATGC
FvANS47	GGAGAGTTTAGGCGTGGGAAAGC	TGAGGCACCAAACCAACGGATG
FvANS51	CGGTATCGCTCTGTTACTGCTAAGG	CAAGGAAAGGGTTCGGTCGAGATG
FvANS52	GTGAAGCGGTCGCAGTACAGAG	GATGACCAGAGCACCACGGATG
FvANS62	GCTGTGAGGACTATGGCTTGTGAG	GGGTCTGTGTGCTCTCCAAATCC
FvANS63	TTACGCCTCATTCAGATGCCTCAG	ACGCTTCAGGAAGTGGCTTAACAG
FvANS66	ACACCAGCGAGGAGCCTTACC	GCCACATGAGGTCCCTGAACTTC
FvANS67	CCCGCAGCCTGAATTGACCATC	TGGAAGAAGCAACCCACAGAAACTC
FvANS69	TCTTGAGGAGCCTTGTAAGCAATGG	CCAGTCGGAGTAAGGGCAGGAG
FvANS71	GAGTCTCTTGGGCTAAAGCGAAGTC	TTGTGGGCAAGCAGGATAGTAATGG
FvANS73	CCAGGGTCAGCCACTCTCTCC	CTACACCGATGCCAGTCCGATTAG
FvANS76	GCGGCTTCCTCACTGTCCTTC	CCTGTGCTCTACACTCCTGAATCTG
FvANS78	CATCATCCACCACATCCGTACAAGG	TGCCAAGGCTTGCTCAAGTCAC
FvANS79	GCTCCTAATCCACCCAAACCAGAAG	AATCCCACCTCCATCACATGCTTTG
FvANS82	CACCAAGCCTGTAGTGGATCATACC	ATTGTGAGGAACGCTGGATCTGTG
FvANS83	GAGGCGTGCTGAGGTGATTGAC	GCGATACATGGTACTGCCAGAGAAG
FvANS85	ACCGCTCCTATACCATCCTCTAGTG	GCATGAAGAAACCCTGGGCTACC
FvANS87	CACTCGGACTGGCTGTTCATACTG	GCCCTTCACTCCCTCTGTTTGC
FvANS88	GCGGCGTGGAACAAGGAGATC	GGCAGGGCGGGTAGTAGTGTC
FvANS89	GTGCTTTCTTCGCTTGAATCGCTAC	AATATCGTTCCGTCTTCTCGTTGGC
FvANS91	TCAGGGAGGAAGTGGGTGAGTATTG	GTCCAGGCAAGCCGTAAGTAAGC
FvANS92	TCGAGTTGTTGTCTGAAGCTCTTGG	CGGGCACAGGAGGTACATCAAAC
FvANS99	CAGCAGAAGAAGCCGAGAAGTAGC	TCAGGGCAAGGTGGGTAGTAGTTC
FvANS104	ATTGTTGGAGGTCTGGAAGTGATGG	ATTGAGATACGAACGGCGGCTTC
FvANS105	GAGGTTCCAAACACATCCCGAGTTC	GGCTGAGGACAAGGTGGGTAGTAG
FvANS106	CGGAGGAGATGAAGGTGGAGGAG	TGGTTGCGGACAGTAGGGATAGC
FvANS109	TCCAACGACCCGAGCACCTATG	GACAGCATCTTCACCAGTCTTCCAC

FvANS111	TGGTCCGACGCACTGGTTCTC	CCTTGTATCCCTTTGGTGTGGTTGG
FvANS114	AGAAGTGACCAACGGCTTGAAGG	GCTCCTCTGCAACTCTCCTAACATC
FvANS121	TTCCTTCCTCTCTACGAGTCCTTGG	GCCGCTCCACGCCATAATTCTC
FvANS122	CCCAAACTCCCTCTCCATGAAAGC	TCATAACACTTCTCCACGCCATAGC
FvANS128	GGCATCCTCACTCTTCTCCTTCAAG	TTGGCAAAGTCTGTGTCCCTGTAAC
FvANS129	AGGTGATGGCTGTGAATTGCTATCC	TGGAGCCGTAATCTGAATGTGAAGG
FvGAPDH	CATTCATCACCACCGACTACA	GAAGGGTCTTCTCATCCTTGAC

Supplementary Table S2. The physicochemical properties of *FvANS* in strawberries.

Gene name	Accession number	Amino acid numbers(aa)	Molecular weight (Da)	pI	Instability Index	Aliphatic Index	Hydropathicity
FvANS1	*FvH4_1g00520.t1*	311	35156.51	5.58	29.38	77.11	-0.421
FvANS2	*FvH4_1g02111.t1*	359	40328.10	5.97	43.24	87.33	-0.324
FvANS3	*FvH4_1g02112.t1*	339	38426.00	5.37	43.18	87.61	-0.291
FvANS4	*FvH4_1g02120.t1*	339	38701.36	5.24	42.98	91.36	-0.345
FvANS5	*FvH4_1g02121.t1*	354	40771.94	5.33	49.93	86.16	-0.391
FvANS6	*FvH4_1g02150.t1*	355	40623.73	4.83	48.28	94.39	-0.275
FvANS7	*FvH4_1g02151.t1*	354	40680.58	4.93	48.38	91.69	-0.317
FvANS8	*FvH4_1g02152.t1*	353	40791.88	4.78	44.55	90.79	-0.259
FvANS9	*FvH4_1g02160.t1*	265	31220.56	4.95	51.17	75.02	-0.513
FvANS10	*FvH4_1g04200.t1*	355	39947.51	6.19	39.02	85.10	-0.253
FvANS11	*FvH4_1g05410.t1*	372	41691.52	6.10	37.12	86.24	-0.289
FvANS12	*FvH4_1g06010.t1*	337	38134.34	5.39	36.84	84.75	-0.331
FvANS13	*FvH4_1g07181.t1*	305	34561.97	5.29	37.02	76.13	-0.556
FvANS14	*FvH4_1g07190.t1*	307	35042.92	5.61	33.66	75.31	-0.535
FvANS15	*FvH4_1g09830.t1*	297	33103.08	5.31	38.78	93.84	-0.128
FvANS16	*FvH4_1g11810.t1*	364	41080.80	5.52	41.11	80.88	-0.476
FvANS17	*FvH4_1g16460.t1*	321	35935.26	5.47	22.87	80.16	-0.303
FvANS18	*FvH4_1g26760.t1*	345	37962.26	5.93	47.89	83.36	-0.237
FvANS19	*FvH4_2g03190.t1*	239	26385.11	4.93	42.11	94.64	-0.226
FvANS20	*FvH4_2g03200.t1*	608	67909.56	5.15	40.97	92.83	-0.208
FvANS21	*FvH4_2g03210.t1*	605	68294.08	5.22	38.91	92.64	-0.238
FvANS22	*FvH4_2g03220.t1*	187	20650.66	5.91	35.96	90.64	-0.260

FvANS23	*FvH4_2g07071.t1*	326	37682.73	5.76	34.68	77.73	-0.695
FvANS24	*FvH4_2g07080.t1*	279	31740.99	5.38	42.07	83.12	-0.556
FvANS25	*FvH4_2g07081.t1*	353	40681.17	5.33	38.28	80.00	-0.594
FvANS26	*FvH4_2g07082.t1*	351	40909.63	5.70	32.52	81.57	-0.504
FvANS27	*FvH4_2g11330.t1*	258	28908.86	5.08	38.75	95.50	-0.267
FvANS28	*FvH4_2g15310.t1*	348	39232.33	5.44	48.80	81.15	-0.348
FvANS29	*FvH4_2g15312.t1*	151	17122.77	9.65	41.77	75.50	-0.481
FvANS30	*FvH4_2g21630.t1*	344	39115.70	5.61	35.61	88.11	-0.280
FvANS31	*FvH4_2g21690.t1*	342	38646.97	6.83	39.97	82.87	-0.361
FvANS32	*FvH4_2g21772.t1*	325	36837.41	5.47	42.38	100.71	-0.181
FvANS33	*FvH4_2g21773.t1*	337	38199.71	5.47	42.31	99.14	-0.158
FvANS34	*FvH4_2g21774.t1*	342	38275.81	6.00	45.18	89.74	-0.278
FvANS35	*FvH4_2g21830.t1*	361	40802.40	5.93	42.17	86.37	-0.372
FvANS36	*FvH4_2g21870.t1*	224	25096.23	5.74	48.63	77.86	-0.324
FvANS37	*FvH4_2g21890.t1*	365	41210.87	6.51	30.86	88.38	-0.354
FvANS38	*FvH4_2g21910.t1*	378	42861.15	5.71	47.95	82.51	-0.337
FvANS39	*FvH4_2g26440.t1*	335	38227.56	5.41	38.33	84.36	-0.529
FvANS40	*FvH4_2g26480.t1*	289	32741.61	5.17	50.30	90.35	-0.329
FvANS41	*FvH4_2g27140.t1*	342	38594.17	5.55	33.99	85.79	-0.205
FvANS42	*FvH4_2g27800.t1*	303	34153.67	4.93	31.38	83.00	-0.426
FvANS43	*FvH4_2g29950.t1*	318	36117.15	5.42	39.43	79.12	-0.383
FvANS44	*FvH4_2g29960.t1*	318	36384.68	5.72	32.78	77.23	-0.316
FvANS45	*FvH4_2g29980.t1*	321	36822.69	5.37	34.98	62.55	-0.441
FvANS46	*FvH4_2g29990.t1*	329	37547.97	5.40	34.38	77.02	-0.311
FvANS47	*FvH4_2g29991.t1*	326	37202.67	4.95	45.22	79.17	-0.256
FvANS48	*FvH4_2g30010.t1*	336	37704.27	6.79	36.84	90.77	-0.212

FvANS49	*FvH4_2g30020.t1*	341	38444.00	5.47	36.90	92.61	-0.167
FvANS50	*FvH4_2g30021.t1*	341	37915.37	5.21	36.32	92.61	-0.123
FvANS51	*FvH4_2g30040.t1*	353	39308.95	6.19	44.28	87.85	-0.122
FvANS52	*FvH4_2g35050.t1*	375	42527.54	7.63	28.65	79.52	-0.308
FvANS53	*FvH4_3g01280.t1*	312	35555.86	5.11	38.40	80.00	-0.385
FvANS54	*FvH4_3g02670.t1*	350	39812.94	5.89	52.31	68.80	-0.486
FvANS55	*FvH4_3g05530.t1*	336	37704.19	6.03	40.76	83.01	-0.213
FvANS56	*FvH4_3g06511.t1*	288	32823.99	5.48	45.25	91.04	-0.247
FvANS57	*FvH4_3g06513.t1*	180	20178.07	5.64	37.16	87.72	-0.349
FvANS58	*FvH4_3g07861.t1*	284	31701.18	5.11	41.49	87.50	-0.285
FvANS59	*FvH4_3g12330.t1*	299	34345.34	5.15	41.10	73.65	-0.400
FvANS60	*FvH4_3g12340.t1*	316	35973.02	4.95	40.65	79.30	-0.283
FvANS61	*FvH4_3g15580.t1*	366	40353.21	5.58	33.72	89.70	-0.211
FvANS62	*FvH4_3g16760.t1*	337	37728.85	6.10	45.49	82.14	-0.291
FvANS63	*FvH4_3g17470.t1*	375	42592.59	5.15	39.08	87.36	-0.358
FvANS64	*FvH4_3g21910.t1*	329	37755.26	6.64	43.42	82.61	-0.316
FvANS65	*FvH4_3g23800.t1*	299	34389.24	5.49	34.88	75.28	-0.428
FvANS66	*FvH4_3g28210.t1*	316	36026.81	5.20	34.59	75.85	-0.433
FvANS67	*FvH4_3g31880.t1*	365	41013.17	6.32	39.00	95.56	-0.208
FvANS68	*FvH4_3g36530.t1*	344	38518.90	5.43	31.18	74.77	-0.309
FvANS69	*FvH4_3g38920.t1*	334	37594.87	6.00	32.93	87.75	-0.300
FvANS70	*FvH4_3g45310.t1*	364	40816.76	5.91	48.55	87.25	-0.273
FvANS71	*FvH4_4g03240.t1*	347	38599.80	5.09	39.38	94.70	-0.228
FvANS72	*FvH4_4g03241.t1*	355	40483.83	5.40	40.43	79.58	-0.467
FvANS73	*FvH4_4g03242.t1*	354	40256.18	7.69	40.75	93.59	-0.216
FvANS74	*FvH4_4g03250.t1*	362	40794.32	5.14	34.07	88.51	-0.332

FvANS75	*FvH4_4g03251.t1*	361	40854.47	5.77	33.68	86.34	-0.365
FvANS76	*FvH4_4g03260.t1*	369	41479.06	5.32	42.24	83.44	-0.363
FvANS77	*FvH4_4g03300.t1*	402	45800.19	6.03	34.77	87.74	-0.380
FvANS78	*FvH4_4g03301.t1*	288	32421.81	5.27	44.87	78.85	-0.394
FvANS79	*FvH4_4g03303.t1*	360	40671.12	5.52	32.83	84.97	-0.396
FvANS80	*FvH4_4g03304.t1*	380	43013.35	6.61	34.92	89.95	-0.334
FvANS81	*FvH4_4g03305.t1*	366	40798.57	5.41	32.00	93.72	-0.165
FvANS82	*FvH4_4g03310.t1*	294	33431.09	5.21	37.38	91.46	-0.288
FvANS83	*FvH4_4g03323.t1*	361	41427.60	5.78	47.38	84.74	-0.331
FvANS84	*FvH4_4g14690.t1*	256	28359.51	4.94	29.05	95.55	-0.035
FvANS85	*FvH4_4g17720.t1*	365	40986.00	7.62	37.52	87.01	-0.335
FvANS86	*FvH4_4g20730.t1*	319	35014.95	5.90	40.87	82.48	-0.159
FvANS87	*FvH4_4g25590.t1*	339	39053.48	5.92	32.91	87.40	-0.417
FvANS88	*FvH4_4g33740.t1*	362	39661.35	5.56	36.92	89.09	-0.194
FvANS89	*FvH4_4g36510.t1*	335	37769.86	5.48	51.48	86.15	-0.269
FvANS90	*FvH4_5g01170.t1*	383	42861.12	5.48	46.41	92.17	-0.330
FvANS91	*FvH4_5g10430.t1*	345	39074.61	5.74	41.35	88.99	-0.323
FvANS92	*FvH4_5g19290.t1*	312	35234.04	4.77	40.15	93.01	-0.186
FvANS93	*FvH4_5g19300.t1*	483	54666.00	8.96	69.78	68.22	-0.689
FvANS94	*FvH4_5g19310.t1*	180	20139.11	7.20	37.47	92.56	-0.320
FvANS95	*FvH4_5g19321.t1*	285	31848.09	5.20	44.99	85.82	-0.324
FvANS96	*FvH4_5g19520.t1*	395	44124.83	6.39	45.58	89.04	-0.083
FvANS97	*FvH4_5g19970.t1*	358	40517.57	6.13	43.30	87.40	-0.278
FvANS98	*FvH4_5g20420.t1*	312	34710.37	5.59	31.19	88.43	-0.215
FvANS99	*FvH4_5g20421.t1*	365	40886.68	5.97	36.53	81.78	-0.306
FvANS100	*FvH4_5g20430.t1*	355	39778.23	5.75	35.86	82.20	-0.304

FvANS101	*FvH4_5g20431.t1*	357	40197.75	5.60	36.63	83.36	-0.316
FvANS102	*FvH4_5g20432.t1*	357	40008.44	5.49	34.82	82.55	-0.318
FvANS103	*FvH4_5g21360.t1*	542	61435.81	5.35	42.50	81.64	-0.402
FvANS104	*FvH4_5g26041.t1*	171	19022.78	5.55	44.47	91.75	-0.100
FvANS105	*FvH4_5g33280.t1*	348	39301.64	5.13	36.86	80.78	-0.341
FvANS106	*FvH4_5g35660.t1*	369	41255.88	5.85	32.74	85.91	-0.321
FvANS107	*FvH4_5g38040.t1*	357	39577.12	5.25	39.83	84.76	-0.245
FvANS108	*FvH4_5g38050.t1*	355	39316.79	5.25	47.02	85.24	-0.225
FvANS109	*FvH4_6g11360.t1*	357	40236.15	6.27	45.27	83.22	-0.354
FvANS110	*FvH4_6g13281.t1*	166	18588.46	7.79	38.39	87.47	-0.356
FvANS111	*FvH4_6g13290.t1*	364	40873.78	5.43	43.71	90.82	-0.326
FvANS112	*FvH4_6g13300.t1*	365	41496.31	5.30	47.68	92.03	-0.318
FvANS113	*FvH4_6g13310.t1*	222	24976.62	6.14	41.63	88.69	-0.320
FvANS114	*FvH4_6g13322.t1*	355	40120.96	5.74	35.31	91.94	-0.350
FvANS115	*FvH4_6g17160.t1*	477	54548.55	6.35	54.04	86.79	-0.230
FvANS116	*FvH4_6g19280.t1*	369	41416.18	5.83	35.81	89.76	-0.327
FvANS117	*FvH4_6g19281.t1*	308	34494.18	5.67	35.41	85.06	-0.321
FvANS118	*FvH4_6g19290.t1*	208	23069.47	5.17	33.22	98.41	-0.057
FvANS119	*FvH4_6g27770.t1*	340	38439.03	5.32	46.09	86.24	-0.363
FvANS120	*FvH4_6g28170.t1*	366	41096.17	5.45	41.19	91.28	-0.283
FvANS121	*FvH4_6g30750.t1*	253	29262.36	5.38	38.72	74.31	-0.373
FvANS122	*FvH4_6g30751.t1*	320	36478.29	5.48	31.79	75.53	-0.400
FvANS123	*FvH4_6g30760.t1*	316	36040.06	5.72	33.42	82.34	-0.384

FvANS124	*FvH4_6g30780.t1*	358	40063.75	6.62	48.41	88.77	-0.227
FvANS125	*FvH4_6g39151.t1*	348	38753.67	5.49	46.41	85.46	-0.373
FvANS126	*FvH4_6g42090.t1*	320	36233.31	5.08	31.46	80.75	-0.409
FvANS127	*FvH4_6g42700.t1*	316	36088.89	5.19	36.43	74.62	-0.438
FvANS128	*FvH4_7g06780.t1*	384	43322.95	5.47	47.45	80.16	-0.393
FvANS129	*FvH4_7g10320.t1*	208	23307.68	5.08	36.89	93.17	-0.275
FvANS130	*FvH4_7g10930.t1*	167	19272.68	5.97	30.02	80.54	-0.503
FvANS131	*FvH4_7g12600.t1*	379	42918.84	5.34	28.38	78.42	-0.268
FvANS132	*FvH4_7g12610.t1*	375	42258.84	5.82	32.93	79.20	-0.351
FvANS133	*FvH4_7g14240.t1*	339	38518.66	5.92	44.51	75.28	-0.384
FvANS134	*FvH4_7g15780.t1*	316	35913.63	5.72	40.39	69.37	-0.491
FvANS135	*FvH4_7g17840.t1*	345	38965.13	5.79	39.05	86.72	-0.362
FvANS136	*FvH4_7g24550.t1*	359	40561.41	6.32	40.75	82.81	-0.389
FvANS137	*FvH4_7g26820.t1*	354	39286.60	5.63	33.02	79.86	-0.288
FvANS138	*FvH4_7g28670.t1*	373	42457.11	5.52	35.14	70.05	-0.449
FvANS139	*FvH4_7g30770.t1*	345	38556.63	5.23	38.40	77.45	-0.381
FvANS140	*FvH4_7g31470.t1*	401	45397.51	6.44	35.56	90.42	-0.259
FvANS141	*FvH4_7g32980.t1*	338	38619.84	5.84	44.46	83.58	-0.436

Supplementary Table S3. The secondary structure prediction of FvANS proteins.

protein	Alpha helix	Random coil	Extended strand
FvANS1	26.37%	53.05%	20.58%
FvANS2	31.75%	44.85%	23.40%
FvANS3	38.35%	44.25%	17.40%
FvANS4	35.99%	46.02%	17.99%
FvANS5	36.72%	48.02%	15.25%
FvANS6	37.75%	47.89%	14.37%
FvANS7	42.66%	44.35%	12.99%
FvANS8	33.99%	47.88%	18.13%
FvANS9	33.96%	50.57%	15.47%
FvANS10	29.86%	52.11%	18.03%
FvANS11	34.68%	49.19%	16.13%
FvANS12	37.09%	47.48%	15.43%
FvANS13	33.44%	48.20%	18.36%
FvANS14	32.57%	51.14%	16.29%
FvANS15	38.72%	43.77%	17.51%
FvANS16	30.77%	48.35%	20.88%
FvANS17	37.07%	49.22%	13.71%
FvANS18	28.70%	57.68%	13.62%
FvANS19	43.10%	46.44%	10.46%
FvANS20	45.39%	40.62%	13.98%
FvANS21	48.10%	38.35%	13.55%
FvANS22	28.34%	56.15%	15.51%
FvANS23	29.45%	54.60%	15.95%
FvANS24	31.18%	55.20%	13.62%
FvANS25	28.05%	51.84%	20.11%
FvANS26	33.90%	45.87%	20.23%
FvANS27	19.38%	58.91%	21.71%
FvANS28	24.71%	53.16%	22.13%
FvANS29	33.11%	49.67%	17.22%
FvANS30	29.07%	47.67%	23.26%
FvANS31	32.16%	47.08%	20.76%
FvANS32	31.08%	48.62%	20.31%
FvANS33	32.94%	45.99%	21.07%
FvANS34	30.12%	51.17%	18.71%
FvANS35	33.24%	49.31%	17.45%
FvANS36	35.71%	49.11%	15.18%
FvANS37	38.36%	48.22%	13.42%
FvANS38	41.53%	46.03%	12.43%
FvANS39	29.25%	52.54%	18.21%
FvANS40	39.45%	42.56%	17.99%
FvANS41	29.53%	52.05%	18.42%
FvANS42	31.68%	50.83%	17.49%

FvANS43	41.51%	45.60%	12.89%
FvANS44	37.74%	46.23%	16.04%
FvANS45	32.71%	50.78%	16.51%
FvANS46	34.35%	48.94%	16.72%
FvANS47	36.81%	49.08%	14.11%
FvANS48	31.55%	52.08%	16.37%
FvANS49	36.66%	47.80%	15.54%
FvANS50	39.30%	47.21%	13.49%
FvANS51	37.39%	48.16%	14.45%
FvANS52	39.20%	48.27%	12.53%
FvANS53	39.10%	45.51%	15.38%
FvANS54	35.71%	50.00%	14.29%
FvANS55	31.55%	46.13%	22.32%
FvANS56	36.81%	47.22%	15.97%
FvANS57	27.78%	50.00%	22.22%
FvANS58	41.90%	46.48%	11.62%
FvANS59	40.13%	46.82%	13.04%
FvANS60	42.41%	46.20%	11.39%
FvANS61	32.51%	48.63%	18.85%
FvANS62	29.08%	48.66%	22.26%
FvANS63	39.47%	46.93%	13.60%
FvANS64	36.17%	44.68%	19.15%
FvANS65	38.13%	46.49%	15.38%
FvANS66	31.96%	52.22%	15.82%
FvANS67	38.08%	40.82%	21.10%
FvANS68	23.55%	57.56%	18.90%
FvANS69	21.56%	59.58%	18.86%
FvANS70	36.54%	47.25%	16.21%
FvANS71	32.28%	52.16%	15.56%
FvANS72	29.58%	54.93%	15.49%
FvANS73	41.81%	47.18%	11.02%
FvANS74	30.11%	56.63%	13.26%
FvANS75	32.13%	55.40%	12.47%
FvANS76	26.56%	56.64%	16.80%
FvANS77	35.82%	48.51%	15.67%
FvANS78	32.29%	55.56%	12.15%
FvANS79	34.44%	49.44%	16.11%
FvANS80	38.16%	43.68%	18.16%
FvANS81	38.52%	48.36%	13.11%
FvANS82	36.73%	44.56%	18.71%
FvANS83	40.17%	45.98%	13.85%
FvANS84	43.75%	41.41%	14.84%
FvANS85	32.60%	54.25%	13.15%
FvANS86	29.78%	52.04%	18.18%

FvANS87	33.33%	48.67%	17.99%
FvANS88	26.52%	53.31%	20.17%
FvANS89	36.72%	51.34%	11.94%
FvANS90	39.95%	45.69%	14.36%
FvANS91	39.13%	47.25%	13.62%
FvANS92	39.42%	50.64%	9.94%
FvANS93	26.09%	62.11%	11.80%
FvANS94	25.56%	59.44%	15.00%
FvANS95	35.79%	50.18%	14.04%
FvANS96	33.42%	47.34%	19.24%
FvANS97	36.03%	49.16%	14.80%
FvANS98	32.69%	44.23%	23.08%
FvANS99	33.15%	47.95%	18.90%
FvANS100	40.56%	43.94%	15.49%
FvANS101	42.58%	41.46%	15.97%
FvANS102	34.73%	48.46%	16.81%
FvANS103	42.80%	43.91%	13.28%
FvANS104	32.75%	54.97%	12.28%
FvANS105	38.79%	45.11%	16.09%
FvANS106	35.50%	50.68%	13.82%
FvANS107	37.25%	47.90%	14.85%
FvANS108	38.03%	47.61%	14.37%
FvANS109	26.61%	53.78%	19.61%
FvANS110	18.07%	55.42%	26.51%
FvANS111	37.64%	47.80%	14.56%
FvANS112	33.97%	49.32%	16.71%
FvANS113	33.33%	47.30%	19.37%
FvANS114	31.83%	44.79%	23.38%
FvANS115	35.22%	48.85%	15.93%
FvANS116	32.79%	50.14%	17.07%
FvANS117	34.74%	49.35%	15.91%
FvANS118	37.98%	43.27%	18.75%
FvANS119	28.53%	49.12%	22.35%
FvANS120	32.79%	48.09%	19.13%
FvANS121	32.91%	51.44%	15.65%
FvANS122	34.69%	54.06%	11.25%
FvANS123	34.49%	51.27%	14.24%
FvANS124	29.05%	53.91%	17.04%
FvANS125	26.44%	53.16%	20.40%
FvANS126	38.12%	45.00%	16.88%
FvANS127	31.65%	52.85%	15.51%
FvANS128	27.34%	56.51%	16.15%
FvANS129	23.08%	52.88%	24.04%
FvANS130	32.93%	48.50%	18.56%

FvANS131	36.41%	50.66%	12.93%
FvANS132	31.73%	49.60%	18.67%
FvANS133	30.38%	48.97%	20.65%
FvANS134	25.63%	55.70%	18.67%
FvANS135	24.35%	54.49%	21.16%
FvANS136	24.79%	56.82%	18.38%
FvANS137	38.42%	50.56%	11.02%
FvANS138	38.07%	46.38%	15.55%
FvANS139	42.61%	46.67%	10.72%
FvANS140	42.89%	43.64%	13.47%
FvANS141	34.02%	44.97%	21.01%
